# Coxsackie and adenovirus receptor is a novel regulator of inflammatory response in endotoxin-induced failing heart.

**DOI:** 10.1016/j.jmccpl.2025.100496

**Published:** 2025-11-06

**Authors:** Reo Matsumura, Mototsugu Nishii, Haruya Usuku, Masahiro Nakayama, Masaki Hachisuka, Naho Misawa, Ryo Saji, Fumihiro Ogawa, Alan Valaperti, Yoshihiro Ishikawa, Ichiro Takeuchi

**Affiliations:** a Department of Emergency Medicine, Yokohama City University Graduate School of Medicine, Yokohama, Japan; b Department of Immunology, University Hospital Zurich, Zurich, Switzerland; c Cardiovascular Research Institute, Yokohama City University Graduate School of Medicine, Yokohama, Japan

**Keywords:** Coxsackie and adenovirus receptor, Endotoxin, Cardiac dysfunction, Inflammatory cytokine, p38 MAPK

## Abstract

The regulatory mechanisms for inflammatory response in the heart to endotoxin, which causes severe cardiac dysfunction, are not fully understood. We hypothesized the involvement of coxsackie and adenovirus receptor (CXADR), which can promote tissue inflammation by potentiating cell-cell adhesion, independent of viral infection, and examined the role of CXADR in endotoxin-induced cardiac dysfunction and its mechanism using an experimental mouse model. Conditional whole-body and endothelium-specific CXADR knockout (W-KO and *E*-KO, respectively) mice were generated using the Cre-loxP system and administered lipopolysaccharide (LPS) or vehicle alone, like wild-type (WT) mice. Cardiac CXADR increased 12 h after LPS challenge in WT mice, along with improved cardiac dysfunction and reduced cardiac expression of interleukin (IL)-6 and IL-1β. Moreover, W-KO in adult mice worsened cardiac dysfunction and increased expression of these cytokines. Meanwhile, *E*-KO exhibited the opposite effects, concomitantly reducing myocardial inflammation. Bulk RNA sequencing analysis identified an enriched IL-17 A signaling pathway capable of inducing IL-6 and IL-1β expression in the heart 12 h after LPS challenge. In this heart, *E*-KO attenuated phosphorylation of p38 but not of upstream mitogen-activated protein kinase kinase (MKK)3/6. Conversely, W-KO augmented phosphorylation of p38, MKK3/6, and NF-κB/p65, which are key drivers of the IL-17 A signaling. Our study is the first to demonstrate that increased CXADR expression plays a dual role as both a pro-inflammatory mediator and an anti-inflammatory protector in endotoxin-induced cardiac dysfunction, possibly by positively or negatively regulating p38 activation depending on its cellular origin. Targeted manipulation of CXADR expression may provide clinical benefits.

## Highlights

• Expression of CXADR is increased in endotoxin-stressed heart.
• IL-17A-mediated inflammatory response underlies endotoxin-induced failing heart.
• CXADR plays dual pro- and anti-inflammatory roles in the heart.
• CXADR diversely regulates the MKK3/6-p38 pathway in the heart.
• Targeted manipulation of CXADR expression may provide clinical benefits.

## Graphical abstract

**Figure:**
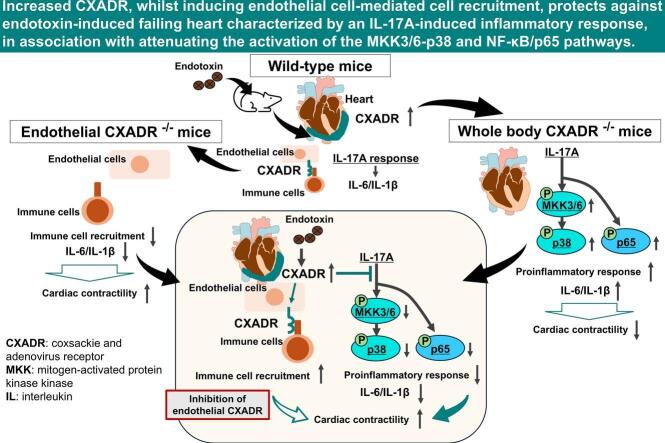

## 1 Introduction

Lipopolysaccharide (LPS), a Gram-negative bacterial endotoxin, mediates innate immune response [1], which causes lethal sepsis with cardiac dysfunction and shock [[2], [3], [4]]. The incidence of cardiac dysfunction in sepsis is as high as 60 %, which drastically reduces survival rates [5,6]. So far, therapies for endotoxin-induced cardiac dysfunction have been proposed, but have shown either no effect or weak results [[7], [8], [9]].

Endotoxin is a major inducer of inflammatory cytokines, including tumor necrosis factor-α, interleukin (IL)-1β and IL-6. Such inflammatory cytokines mediate myocardial depression, and play a pathological role in endotoxin-induced cardiac dysfunction [10]. These incriminating factors are generally considered to be paracrine circulating molecules produced as a consequence of the systemic inflammatory response to endotoxin. Meanwhile, they can also be synthesized by endotoxin-stressed cardiac tissue, including cardiomyocytes, cardiac fibroblast, endothelial cells, and even infiltrating immune cells [[11], [12], [13], [14]]. Systemic anti-inflammatory therapies have failed to improve mortality in patients with severe sepsis [8,15]. This may suggest the pathological importance of tissue-level local inflammatory response to endotoxin. However, its regulatory mechanisms are not fully understood.

The coxsackievirus and adenovirus receptor (CXADR), that originally emerged as a receptor for coxsackievirus and adenovirus [16], has been shown to be essential for the early embryonic development of heart and rapidly reduce following birth [17]. However, increased expression of CXADR has been observed in the failing hearts derived from cardiomyopathy, autoimmune myocarditis, and myocardial infarction [[18], [19], [20]]. CXADR, which is expressed on cardiomyocytes, endothelial cells, and epithelial cells, has been shown to act as a cell-cell adhesion molecule [18,20,21,22,23,24,25]. To date, *vivo* functional analysis with genetically modified mice has demonstrated that, in addition to controlling tissue homeostasis [26], CXADR-mediated cell-cell adhesion contributes to tissue inflammation and dysfunction under stressful conditions, independent of viral infection [[27], [28], [29]]. Particularly, mice with cardiomyocyte-specific overexpression of CXADR developed severe cardiomyopathy, manifested by disorganization and degeneration of the cardiomyocytes, in association with activation of the beta-catenin signaling pathway and/or disruption of the adherens junctions, and died by four weeks. Conversely, administration of doxycycline to turn off transgene expression rescued mice from this advanced cardiomyopathic phenotype [27]. Moreover, this specific overexpression also induced myocardial inflammation unrelated to viral infection [28].

Thus, we focused on the CXADR as a promising therapeutic target for inflammatory cardiac dysfunction and hypothesized that increased CXADR may be involved in the pathological inflammatory response in the heart to endotoxin, which causes cardiac dysfunction. To test this hypothesis, we examined the expression of CXADR and its role in endotoxin-induced cardiac dysfunction as well as the potential mechanisms, using an experimental mouse model. Unexpectedly, our data demonstrated an anti-inflammatory protective role of increased CXADR in endotoxin-induced failing heart.

## 2 Methods

### 2.1 Ethics statement

All animal experiments were approved by the Institutional Animal Care and Use Committee of Yokohama City University (license number: F-A-20-081) and conducted following university guidelines. Experiments with genetically modified mice were also approved by the Institutional Biosafety Committee for Recombinant DNA Experiments at Yokohama City University (license number: F-D-20-23).

### 2.2 Conditional CXADR knockout mice and experimental model

Endothelium-specific knockout (*E*-KO), whole-body knockout (W-KO), and each control (E-CT and W-CT) mice were generated, as shown in Figs. S1(A) and (B). All experiments were conducted using eight- to ten-week-old female mice, as a stronger innate immune response has been reported to be induced after endotoxin challenge in female mice than in male mice [30].

For *E*-KO mice, CXADR flox mice (B6;129S2-Cxadrtm1.1Ics/J; stock No. 017359) from Jackson Laboratory were crossed with Tek-Cre transgenic mice (B6.Cg-Tg (Tek-cre) 1Ywa; stock No. RBRC04495) from RIKEN. CXADR flox mice had loxP sites flanking exon 2, and Tek-Cre mice expressed Cre recombinase under the endothelial cell-specific Tek promoter.

For W-KO mice, CXADR flox and Cre-ERT mice (B6.Cg-Tg (CAG-cre/Esr1*) 5Amc/J; stock No. 004682) from Jackson Laboratory were backcrossed with BALB/cAnNCrlCrlj for six generations before crossing. To circumvent the embryonic lethality, W-KO mice were generated using expression of the tamoxifen-inducible mutant estrogen receptor fusion protein [31]. Tamoxifen (Nacalai Tesque, Cat. #: 19885–34) was prepared at 20 mg/mL in corn oil and administered intraperitoneally at 75 mg/kg daily for five days.

CXADR flox/flox mice, littermates of *E*-KO and W-KO mice, were used for E-CT and W-CT mice, with tamoxifen administered to W-CT mice as well. Genotyping was performed to confirm CXADR deletion, and primers are listed in Table S1. Mice were blinded after genotyping and housed under standard conditions (12/12-h light/dark cycle, 24 ± 1 °C temperature, 55 ± 5 % humidity) with free access to food and water.

Ultimately, to produce an experimental endotoxin-induced failing heart model, eight- to ten-week-old female conditional CXADR knockout mice, as well as the control mice and wild-type (WT) mice, were injected intraperitoneally with O111:B4 LPS (8, 20, or 32 mg/kg) from *Escherichia coli* (Sigma-Aldrich, Cat. #: L2630), as previously reported [30,32]. Preliminarily, doses of LPS were determined based on the onset of cardiac dysfunction and a survival rate. Sham controls were similarly injected with phosphate-buffered saline (PBS).

### 2.3 Echocardiography

*trans*-Thoracic echocardiography was performed pre- and 6 and 12 h post-challenge using a Vevo 3100LT system (VisualSonics, Fujifilm) with an MX400 20–46 MHz linear array transducer. Heart rate (HR) was monitored, and short-axis views of the heart at the papillary muscle level were imaged. M-mode views measured left ventricular (LV) dimensions such as LV diastolic dimension (LVDd) and LV systolic dimension (LVSd). Fractional shortening (FS) was calculated using Vevolab software (VisualSonics, Fujifilm). Echocardiography was performed by the same examiner blinded to group identity.

### 2.4 Blood pressure and rectal temperature measurements

Systolic blood pressure (SBP) and rectal temperature (RT) were measured in conscious mice before and 12 h after challenge using a tail-cuff system (MK-2000, Muromachi Kikai Co.) and a rectal thermometer (AD-1687, A&D Co), respectively.

### 2.5 Isolation and preservation of heart samples

Heart samples were isolated and preserved for RNA, protein, and histological analysis, as we previously described [33].

### 2.6 RNA extraction and quantitative PCR

RNA extraction was conducted using the RNeasy Mini Kit (Qiagen, Cat. #: 74106) as per the manufacturer's protocol. cDNA was synthesized using the High Capacity cDNA Reverse Transcription Kit (Thermo Fisher Scientific, Cat. #: 4374966). Quantitative real-time PCR was performed with TB Green Premix Ex Taq II (Takara, Cat. #: RR820A) using a CFX96 Real-Time PCR Detection System (Bio-Rad). Primers are listed in Table S1, and relative mRNA expression was analyzed using the 2^−ΔΔCt^ method.

### 2.7 Western blot analysis

Proteins were extracted from bulk heart tissues and analyzed by SDS-PAGE and Western blotting. Primary antibodies for CXADR, phosphorylated p38-mitogen-activated protein kinase (MAPK) (Thr180/Tyr182), total p38-MAPK, phosphorylated mitogen-activated protein kinase kinase (MKK) 3/6, total MKK3, total MKK6, phosphorylated p65, total p65, phosphorylated TGF-β-activated kinase1 (Ser412) (TAK1), total TAK1, and GAPDH were from Novus Biologicals (Cat. #: NBP1–88192), Cell Signaling Technology (Cat. #: 9211, 9212, 9236, 8535, and 8550), Santa Cruz Biotechnology (Cat. sc-136,548 and 8008), and Cell Signaling Technology (Cat. #9339, 4505, and 2118), respectively. Secondary antibodies were from Cell Signaling Technology (Cat. #7074 and 7076). Enhanced chemiluminescent images with ECL reagent were captured and quantified with the LAS-3000 luminescent image analyzer (Fujifilm, Co.).

### 2.8 Histological analysis

Heart sections were stained with Hematoxylin and Eosin (H&E) and examined under a light microscope with images captured by a digital camera attached to a BZ-X800 microscope (KEYENCE).

### 2.9 The analysis of RNA sequencing, differential expression, and gene set enrichment

We evaluated the quality of isolated RNA with the TapeStation system using the High Sensitivity RNA ScreenTape (Agilent) (Table S2). Subsequently, Eurofins Co., Ltd. conducted RNA sequencing, generating 150-bp paired-end reads with over 20 million reads per sample on a NovaSeq 6000 (Illumina). The trimmed mean of M-values (TMM)-normalized counts were calculated by the calcNormFactor in the EdgeR R package [34]. Gene expression data were generated by the TMM-normalized counts. Differential expression analysis was done by the glmQLFTest function. The resulting *P*-value was adjusted using Benjamini and Hochberg's approach for controlling the false discovery rate (FDR). Genes were deemed differentially expressed with FDR < 0.05 and a log2 fold change (FC) > 1.0 or 3.0, or < −1.0 or − 3.0.

Enrichment analysis of gene sets from the Gene Ontology Biological Process (GOBP) and the Kyoto Encyclopedia of Genes and Genomes (KEGG) was conducted using StringApp software Version 2.1.1 (https://apps.cytoscape.org/apps/stringapp) in differentially expressed genes (DEGs) with high confidence gene-gene interactions (score = 0.700) [35]. Besides, gene set enrichment analysis was also conducted using the GSEA software Version 4.3.2 (https://www.gsea-msigdb.org/gsea/index.jsp) to enrich gene expression data with ontology gene sets from the Molecular Signature Database (MSigDB) [36]. Gene sets with FDR of <0.01 and the strength of pathway enrichment of >1.0 (StringApp) or with FDR of <0.01 (GSEA) were considered significantly enriched.

### 2.10 Statistical analysis

Data were expressed as mean ± SEM or % for indicated number of mice. Nonparametric Mann-Whitney *U* test or Fisher's exact test was applied for the two independent groups. ANOVA, followed by Dunnett's test and Tukey's HSD test, was applied to identify changes over time, namely pre-treatment, 6 h post-treatment, and 12 h post-treatment. Differences between pre-treatment and 12 h post-treatment were evaluated by the Wilcoxon signed-rank test.  Log-rank test was used to analyze Kaplan-Meier survival curves. JMP ver. 17.0 was used for statistical tests. A *P*-value <0.05 was considered statistically significant. Three biological replicates were performed for each experiment.

## 3 Results

### 3.1 CXADR expression in the heart after the endotoxin challenge

**Fig. 1:**
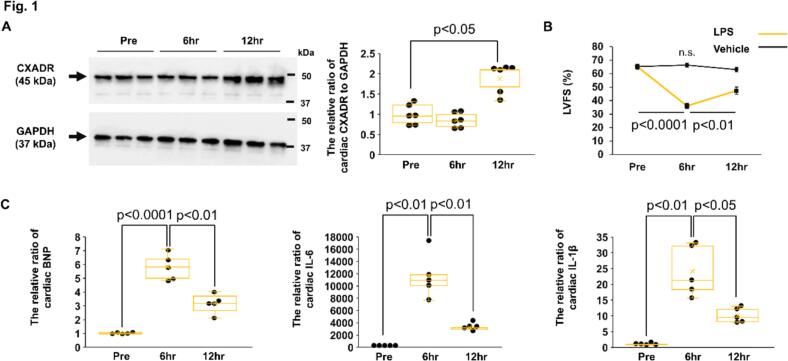
**CXADR expression in the heart after the endotoxin challenge.**. (**A**) Cardiac protein expression of coxsackievirus and adenovirus receptor (CXADR) (*n* = 6 each), (**B**) left ventricular fractional shortening (LVFS) (*n* = 9 each), and (**C**) cardiac mRNA expression of B-type natriuretic peptide (BNP), interleukin (IL)-6, and IL-1β (*n* = 5 each) were assessed by immunoblotting, serial echocardiography, and real-time quantitative PCR, respectively, in wild-type mice before and after challenge with 32 mg/kg of lipopolysaccharide (LPS) or vehicle only. Bulk heart samples for protein and mRNA analysis were collected at pre-challenge, 6 h post-challenge, and 12 h post-challenge. Protein expression was expressed as a relative ratio to GAPDH. Full-length blots of CXADR and GAPDH are presented in Fig. S2. The mRNA expression was expressed as a relative ratio to pre-challenge. Individual data for protein and mRNA expressions are shown as dot plots of the box and whisker, and LVFS is shown as mean (closed squares) ± SEM (error bars). *P* < 0.05, 0.01, or 0.0001 and n.s. ‘not significant’, as determined by two-way ANOVA followed by Dunnett test and Tukey's HSD test. Three biological replicates were performed for each experiment.

The expression of CXADR was examined in the hearts of WT mice. CXADR protein expression significantly increased 12 h after challenge with 32 mg/kg of LPS, but not 6 h after challenge, compared to pre-challenge (The relative ratio to GAPDH: 1.84 ± 0.25 and 0.85 ± 0.11 vs. 1.00 ± 0.17, *P* = 0.0341 and 0.7956, respectively) (Fig. 1(A)). Echocardiographic contractility did not change after vehicle alone challenge (*P* = 0.3975), but reduced 6 h after LPS challenge compared to pre-challenge (LVFS: 36.0 ± 2.1 vs. 65.2 ± 1.9 %, *P* < 0.0001, respectively) and subsequently improved at 12 h (47.4 ± 2.8 %, *P* = 0.0052 vs. 6 h) (Fig. 1(B)). Cardiac mRNA expression of B-type natriuretic peptide (BNP), IL-6, and IL-1β significantly increased 6 h after LPS challenge (The relative ratio to pre-challenge: 5.8 ± 0.95, *P* < 0.0001; 11,596.9 ± 1631.1, *P* = 0.0005; 24.1 ± 3.6, *P* = 0.0012; respectively) but reduced at 12 h compared to 6 h (The relative ratio to pre-challenge: 3.2 ± 0.97, *P* = 0.0059; 3188.8 ± 509.0, *P* = 0.0057; 10.1 ± 1.6, *P* = 0.0278; respectively) (Fig. 1(C)). Collectively, CXADR protein expression increased in endotoxin-stressed hearts, along with recovery of cardiac dysfunction and reduced expression of inflammatory cytokines.

### 3.2 Inactivation of whole-body CXADR exacerbated endotoxin-induced cardiac dysfunction

**Fig. 2:**
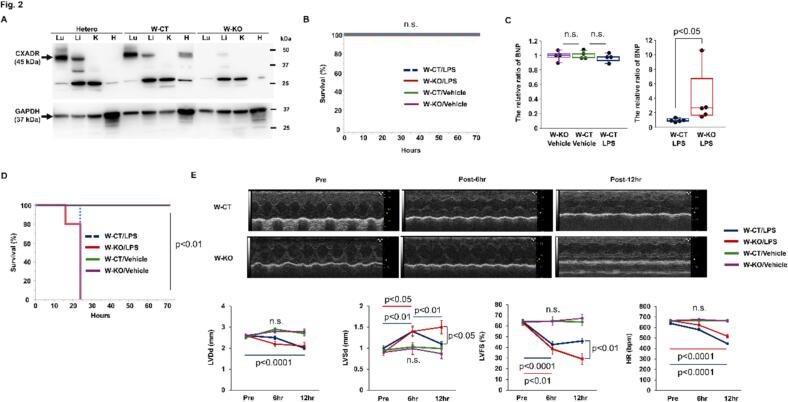
**The detrimental effect of CXADR loss in endotoxin-induced cardiac dysfunction.**. **(A)** Coxsackievirus and adenovirus receptor (CXADR) protein expressions in lung (Lu), liver (Li), kidney (K), and heart (H) of whole-body CXADR knockout (W-KO), control (W-CT), and heterogynous type mice were assessed by immunoblotting. Representative blots of CXADR in each group are shown. Full-length blots of CXADR and GAPDH are presented in Fig. S3. (**B** and **D**) Survival during 72-h observation after challenge with lipopolysaccharide (LPS) at 8 (**B**) or 32 (**D**) mg/kg or vehicle alone was evaluated in W-KO mice (LPS: *n* = 10 each, vehicle alone: n = 10 each) and W-CT mice (LPS: *n* = 11 each, vehicle alone: n = 10 each). *P* < 0.01 vs. vehicle, as determined by the log-rank test. **(C)** The mRNA expression of cardiac B-type natriuretic peptide (BNP) was assessed by real-time quantitative PCR in W-CT and W-KO mice after challenge with 8 mg/kg of LPS or vehicle alone and expressed as a relative ratio to the W-CT mice (*n* = 4 each for left panel; *n* = 5 each for right panel). Bulk heart samples were collected 12 h after challenge. Individual data are shown as dot plots of the box-and-whisker diagram. *P* < 0.05 and n.s. ‘not significant’, as determined by the Mann-Whitney *U* test. (**E)** Serial echocardiographic analyses were performed on W-CT and W-KO mice before and 6 and 12 h after challenge with 32 mg/kg of LPS or vehicle alone. Representative findings and quantitative data (W-KO and W-CT/vehicle alone: n = 5 each, W-KO and W-CT/LPS: *n* = 8 and 14, respectively) are shown in upper panel and lower panel, respectively. Echocardiographic data such as LVDd (left ventricular diastolic dimension), LVSd (LV systolic dimension), FS (fractional shortening), and HR (heart rate) are shown as mean (closed squares) ± SEM (error bars). P < 0.05, 0.01, or 0.0001 and n.s. over time, or P < 0.05 or 0.01 for the two independent groups, as determined by the two-way ANOVA, followed by the Dunnett test and Tukey's HSD test, and Mann-Whitney *U* test, respectively. Three biological replicates were performed for each experiment.

We investigated the role of increased CXADR in endotoxin-induced cardiac dysfunction using W-KO mice and their littermate controls, W-CT mice. Firstly, we confirmed that CXADR protein expression was lost in the heart, as well as in the lung and liver, of W-KO mice compared to W-CT and heterozygous genotype mice (Fig. 2(A)). Next, upon the administration of 8 mg/kg LPS, all W-KO and W-CT mice survived during the 72-h observation, as did the vehicle alone-administered W-KO or W-CT mice (Fig. 2(B)). In this low dose setting, the cardiac mRNA expression of cardiac stress marker BNP did not increase 12 h post-challenge in W-CT mice (*P* = 0.4069 vs. vehicle alone), but significantly increased in W-KO mice (The relative ratio to W-CT mice: 3.9 ± 1.7, *P* = 0.0122) (Fig. 2(C)). Alternatively, after administration of 32 mg/kg LPS, all W-KO and W-CT mice died during the 72-h observation (Survival: 0 % [*n* = 0/10] and 0 % [n = 0/11] vs. sham 100 % [*n* = 5/5], Log-rank test *P* = 0.0006 and 0.0001, respectively) (Fig. 2(D)). In this high dose setting, cardiac function was examined over time, as shown in Fig. 2(E) and Table S3. No significant changes in echocardiographic contractility and dimensions were observed after challenge with vehicle alone in W-KO and W-CT mice. However, 6 h after the LPS challenge, LVSd increased and LVFS reduced in both groups. Subsequently, at 12 h, LVSd reduced in W-CT mice, but not in W-KO mice. Moreover, LVFS was significantly lower in W-KO mice than in W-CT mice. Collectively, these observations suggest that increased CXADR in endotoxin-stressed heart plays a protective role against established cardiac dysfunction.

### 3.3 Endothelium-specific knockout of CXADR ameliorated endotoxin-induced cardiac dysfunction

**Fig. 3:**
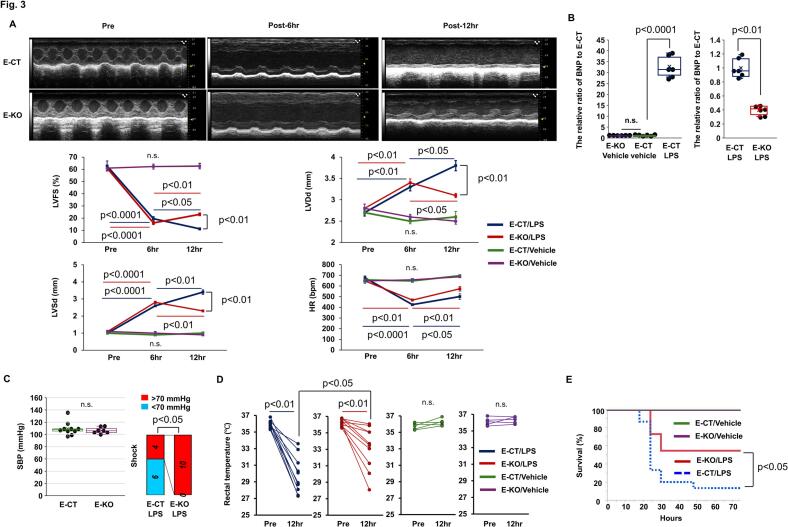
**The protective effect of endothelium-specific CXADR knockout on endotoxin-induced cardiac pathology.**. (**A)** Serial echocardiographic analyses were performed on endothelium-specific coxsackievirus and adenovirus receptor (CXADR) knockout (*E*-KO) and control (E-CT) mice before and 6 and 12 h after challenge with 20 mg/kg of lipopolysaccharide (LPS) or vehicle alone. Representative findings and quantitative data (E-KO and E-CT/vehicle alone: *n* = 5 each, E-KO and E-CT/LPS: *n* = 8 each) are shown in upper panel and lower panel, respectively. Echocardiographic data such as LVDd (left ventricular diastolic dimension), LVSd (LV systolic dimension), FS (fractional shortening), and HR (heart rate) are shown as mean (closed squares) ± SEM (error bars). *P* < 0.05, 0.01, or 0.0001 and n.s. ‘not significant’ over time or *P* < 0.01 for the two independent groups, as determined by the two-way ANOVA, followed by the Dunnett test and Tukey's HSD test, and Mann-Whitney *U* test, respectively. (**B)** The mRNA expression of cardiac B-type natriuretic peptide (BNP) was assessed by real-time quantitative PCR in *E*-KO and E-CT mice after challenge with 20 mg/kg of LPS or vehicle alone and expressed as a relative ratio to *E*-CT mice (*n* = 6 each). Bulk heart samples were collected 12 h after challenge. Individual data are shown as dot plots of the box-and-whisker diagram. *P* < 0.01 or 0.0001 and n.s., as determined by the Mann-Whitney *U* test. (**C**) The differences in systolic blood pressure (SBP) 12 h after vehicle alone administration (left panel, *n* = 5 each), as well as in the shock status indicated by <70 mmHg 12 h after the 20 mg/kg LPS challenge (right panel, *n* = 10 each), were evaluated between the *E*-KO and E-CT mice. n.s. for SBP and *P* < 0.05 for shock, as determined by the Mann-Whitney *U* test and Fisher's exact test, respectively. (**D**) Changes in rectal temperature 12 h after challenge with 20 mg/kg of LPS or vehicle alone were evaluated in the E-KO and E-CT mice (LPS: n = 10 each, vehicle alone: n = 5 each). Individual data are shown as dot plots. P < 0.01 and n.s. over time or P < 0.05 for the two independent groups, as determined by the Wilcoxon signed-rank test or Mann-Whitney *U* test, respectively. **(E)** Survival during 72-h observation after challenge with 20 mg/kg of LPS or vehicle alone was evaluated in *E*-KO (*n* = 11 or 10, respectively) and E-CT (*n* = 15 or 10, respectively) mice. P < 0.05, as determined by the log-rank test. Three biological replicates were performed for each experiment.

We investigated the role of endothelial CXADR in endotoxin-induced cardiac dysfunction using *E*-KO mice and their littermate controls, *E*-CT mice. Changes in echocardiographic parameters after challenge with 20 mg/kg of LPS or vehicle alone were examined, as shown in Fig. 3(A) and Table S4. There were no significant changes in echocardiographic parameters following the vehicle alone challenge in *E*-KO and E-CT mice. However, 6 h after the LPS challenge, LVFS decreased and LVDd and LVSd increased in both groups. Subsequently, at 12 h, these parameters deteriorated further in *E*-CT mice, but improved in E-KO mice. Consistently, 12 h after LPS challenge, increased expression of cardiac BNP (The relative ratio to vehicle alone challenge in *E*-CT mice: 32.78 ± 2.05, *P* < 0.0001) was inhibited in *E*-KO mice (The relative ratio to E-CT mice: 0.40 ± 0.03, *P* = 0.0046) (Fig. 3(B)). Next, we examined the shock state. SBP at baseline did not differ in *E*-KO and E-CT mice (106.4 ± 1.9 vs. 109.4 ± 3.4 mmHg, *P* = 0.6594, respectively) (Fig. 3(C), left panel), but at 6 h after LPS challenge, all mice were unmeasurable (<70 mmHg) and subsequently at 12 h, more E-KO mice recovered from shock than E-CT mice (SBP < 70 mmHg: 0/10 [0 %] vs. 6/10 [60 %], *P* = 0.0108; respectively) (Fig. 3(C), right panel). Additionally, there was no change in RT after vehicle alone challenge in *E*-KO and E-CT mice (*P* = 0.3390 and 0.1133 vs. pre-challenge, respectively), whereas hypothermia was observed 12 h after LPS challenge in *E*-CT mice (RT: 29.9 ± 0.7 °C, *P* = 0.0051 vs. pre-challenge), and improved in *E*-KO mice (RT: 33.2 ± 0.8 °C, *P* = 0.0125 vs. E-CT mice) (Fig. 3(D)). Ultimately, when assessing survival after challenge with LPS, 72-h survival rate was only 13 % [*n* = 2/15] in E-CT mice, but significantly increased to 55 % [*n* = 6/11] in E-KO mice (Log-rank test *P* = 0.0193) (Fig. 3(E)). Collectively, our data suggest that endothelial CXADR contributes to endotoxin-induced cardiac dysfunction as well as shock and hypothermia, which in turn adversely affects survival.

### 3.4 CXADR regulates inflammatory response in endotoxin-induced failing heart

**Fig. 4:**
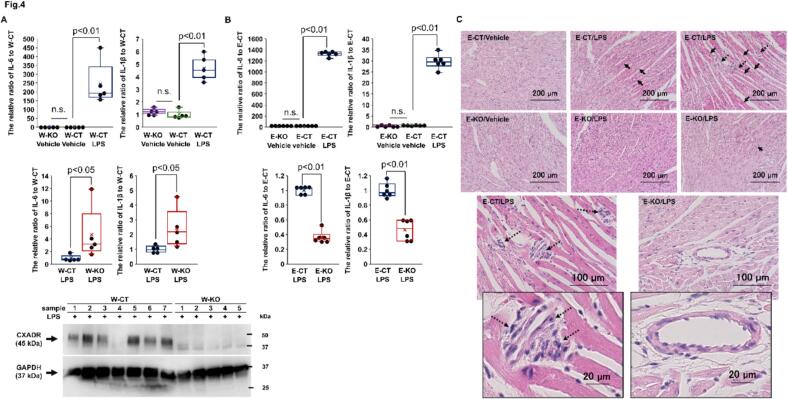
**CXADR regulates inflammatory response in the heart after endotoxin challenge.**. (**A** and **B**) The mRNA expression of cardiac interleukin (IL)-6 and IL-1β was assessed by real-time quantitative PCR in whole-body coxsackievirus and adenovirus receptor (CXADR) knockout (W-KO), endothelium-specific CXADR knockout (*E*-KO), and respective control (W-CT or E-CT) mice after challenge with (**A**) 8 or (**B**) 20 mg/kg of lipopolysaccharide (LPS) or vehicle alone and expressed as a relative ratio to W-CT or E-CT mice (*n* = 5 or 6, respectively). Bulk heart samples were collected 12 h after challenge. Individual data are shown as dot plots of the box-and-whisker diagram. *P* < 0.05 or 0.01 and n.s. ‘not significant’, as determined by the Mann-Whitney *U* test. The protein expression of cardiac CXADR was assessed by immunoblotting in W-CT and W-KO mice after challenge with 8 mg/kg of LPS (*n* = 7 and 5, respectively). Bulk heart samples were collected 12 h after challenge. Full-length blots of CXADR and GAPDH are presented in Fig. S4. **(C)** Histological findings in heart tissue from *E*-KO and E-CT mice after challenge with 20 mg/kg of LPS or vehicle alone were evaluated by hematoxylin-eosin staining [Ten cross-sectional slices from each sample (n = 5 each group)]. Bulk heart samples were collected 12 h after challenge. Closed arrows: acidification of myocardial cytoplasm or disarrangement of fibers; Dotted arrows: mononuclear cell infiltration from microvessels. Representative images of each group are shown. Scale bars: 200, 100, and 20 μm for magnification 200, 400, and 1200×, respectively. Three biological replicates were performed for each experiment.

We examined the role of CXADR on inflammatory response in endotoxin-induced failing heart. Increased cardiac expression of IL-6 and IL-1β in W-CT mice 12 h after LPS challenge (The relative ratio to vehicle alone: 243.1 ± 52.8, *P* = 0.0077; 4.67 ± 0.44, *P* = 0.0077, respectively) was further enhanced by preventing the induction of cardiac CXADR protein expression via W-KO (The relative ratio to W-CT mice: 4.7 ± 1.8, *P* = 0.0212; 2.4 ± 0.6, *P* = 0.0216; respectively) (Fig. 4(A)). Conversely, the increased expression of these cytokines in *E*-CT mice after LPS challenge (The relative ratio to vehicle alone: 1325.2 ± 18.0, *P* = 0.0048; 29.6 ± 1.3, *P* = 0.0048; respectively) was inhibited by the *E*-KO (The relative ratio to E-CT mice: 0.37 ± 0.03, *P* = 0.0047; 0.46 ± 0.06, *P* = 0.0048; respectively) (Fig. 4(B)). Moreover, myocardial injury, indicated by acidification of the myocardial cytoplasm and disarrangement of fibers, and mononuclear cell infiltration around the microvasculature were unevenly scattered in LPS-challenged E-CT hearts, but less prevalent in LPS-challenged E-KO hearts and absent in vehicle alone-challenged hearts (Fig. 4(C)). These results suggest that increased CXADR negatively regulates the inflammatory response in endotoxin-induced failing heart, whilst also inducing it partly through endothelial cells.

### 3.5 The IL-17 A signaling pathway in endotoxin-induced failing heart

To explore the biological signatures underlying endotoxin-induced cardiac dysfunction and identify the involvement of CXADR, bulk heart RNA sequencing data in E–CT and E–KO mice 12 h after challenge with 20 mg/kg of LPS or vehicle alone were integrally analyzed (BioProject: PRJDB19731).

**Fig. 5:**
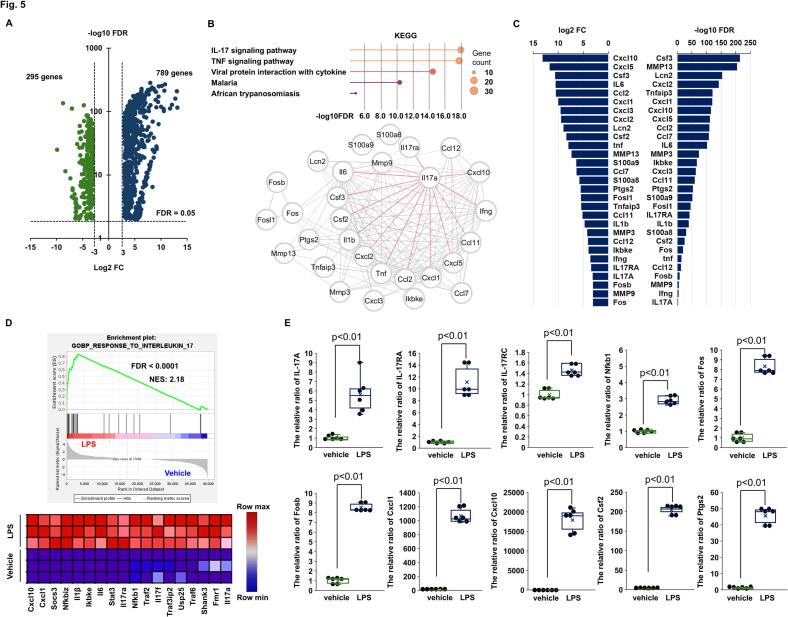
**Bulk heart RNA sequencing in mice after endotoxin challenge.**. (**A)** Volcano plot showing differentially expressed genes (DEGs) after 20 mg/kg lipopolysaccharide (LPS) challenge compared to after vehicle alone challenge (blue: upregulation, green: downregulation, *n* = 3 each). DEGs were identified with a false discovery rate (FDR) of <0.05 and a log2 (fold change: FC) of >3.0 or < −3.0. Bulk heart samples were collected 12 h after challenge. (**B**) The Kyoto Encyclopedia of Genes and Genomes (KEGG) gene set was enriched by up-regulated DEGs with gene-gene interactions using the StringApp software. (**C**) DEGs involved in the enrichment of the IL-17 signaling pathway in the KEGG gene set are shown. (**D**) In the Gene Ontology Biological Process (GOBP) gene set, response to interleukin-17 was enriched by the GSEA software. Significant pathway enrichment was determined by FDR of <0.01. (**E**) Cardiac mRNA expression of cytokines and chemokines was assessed by real-time quantitative PCR in control mice after challenge with 20 mg/kg of LPS or vehicle alone and expressed as a relative ratio to the vehicle alone (*n* = 6 each). Bulk heart samples were collected 12 h after challenge. Individual data are shown as dot plots of the box-and-whisker diagram. *P* < 0.01, as determined by the Mann-Whitney *U* test. Three biological replicates were performed for each experiment. (For interpretation of the references to colour in this figure legend, the reader is referred to the web version of this article.)

In E-CT mice, the expression of 789 genes was significantly upregulated, and 295 genes significantly downregulated after LPS challenge compared to after vehicle alone challenge (Fig. 5(A)). Pathway enrichment analysis was conducted on these DEGs. In up-regulated DEGs, the IL-17 signaling pathway in the KEGG gene set was most significantly enriched, together with the interactions between IL-17 A and inflammatory mediators (Fig. 5(B)). The DEGs involved in its pathway enrichment were IL-17 A, its receptor A (RA), the downstream transcription factor activator protein-1 (AP-1) comprising Fos like 1 (Fosl1), Fos proto-oncogene (Fos), and FosB proto-oncogene (Fosb), and the target genes such as pro-inflammatory cytokines IL-6, IL-1β, and tumor necrosis factor (TNF)-α, C-X-C motif chemokine ligand (Cxcl)-1, 2, 3, 5, and 10, C—C motif chemokine (Ccl)-7, 11, and 12, colony stimulating factor (Csf)-2 and 3, lipocalin 2 (Lcn2), S100 calcium binding protein A (S100a)-8 and 9, matrix metalloproteinase (Mmp)-3, 9, and 13, and prostaglandin-endoperoxide synthase 2 (Ptgs2) (Fig. 5(C)). The GSEA analysis with the GOBP gene set also showed an enriched response to IL-17 (Fig. 5(D)). Moreover, as shown in Fig. 5(E) as well as Fig. 4(B), quantitative PCR confirmed a significant increase in gene expression of the IL-17 signaling pathway after LPS challenge (The relative ratio to vehicle alone challenge: [IL-17 A] 5.6 ± 0.8, *P* = 0.0048; [IL-17RA] 11.1 ± 1.1, *P* = 0.0046; [IL-17RC] 1.5 ± 0.04, *P* = 0.0046; [Nfkb1] 2.9 ± 0.1, *P* = 0.0050; [Fos] 8.3 ± 0.3, *P* = 0.0046; [Fosb] 8.5 ± 0.2, *P* = 0.0046; [Csf2] 204.0 ± 4.8, *P* = 0.0041; [Cxcl1] 1066.3 ± 42.1, *P* = 0.0048; [Cxcl10] 17913.1 ± 1201.3, *P* = 0.0048; [Ptgs2] 45.6 ± 2.0, *P* = 0.0046). Collectively, IL-17 A-mediated inflammatory responses were identified in endotoxin-induced cardiac dysfunction.

### 3.6 Potential involvement of CXADR in IL-17 A-mediated inflammatory responses

**Fig. 6:**
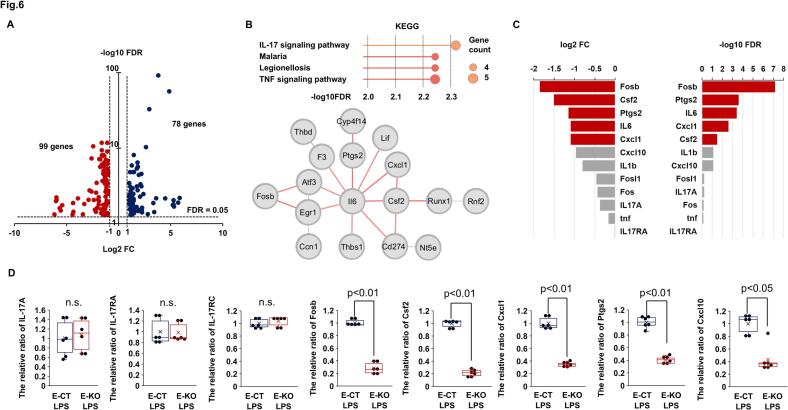
**Endothelial CXADR and the interleukin (IL)-17 response.**. (**A)** Volcano plot showing differentially expressed genes (DEGs) (blue: upregulation, red: downregulation) after challenge with 20 mg/kg of lipopolysaccharide (LPS) in the hearts of endothelium-specific coxsackievirus and adenovirus receptor (CXADR) knockout (*E*-KO) mice compared to the hearts of control (E-CT) mice (*n* = 3 each). Bulk heart samples were collected 12 h after challenge. DEGs were identified with a false discovery rate (FDR) of <0.05 and a log2 (fold change: FC) of >1.0 or < −1.0. (**B**) The Kyoto Encyclopedia of Genes and Genomes (KEGG) gene set was enriched by down-regulated DEGs with gene-gene interactions using the StringApp software. Significant pathway enrichment was determined by FDR of <0.01. (**C**) DEGs involved in the enrichment of the IL-17 signaling pathway in the KEGG gene set are shown. (**D**) Cardiac mRNA expression of cytokines and chemokines was assessed by real-time quantitative PCR in the *E*-CT and E-KO mice after challenge with 20 mg/kg of LPS and expressed as a relative ratio to E-CT mice (*n* = 6 each). Bulk heart samples were collected 12 h after challenge. Individual data are shown as dot plots of the box-and-whisker diagram. *P* < 0.05 and n.s. ‘not significant’, as determined by the Mann-Whitney *U* test. Three biological replicates were performed for each experiment. (For interpretation of the references to colour in this figure legend, the reader is referred to the web version of this article.)

Bulk heart RNA sequencing data showed that the expression of 78 genes was upregulated, and the expression of 99 genes was downregulated 12 h after LPS challenge in *E*-KO hearts compared to E-CT hearts (Fig. 6(A)). In down-regulated DEGs, the IL-17 signaling pathway of the KEGG gene set was most significantly enriched, along with gene-gene interactions including inflammatory mediators and transcription factors Fosb, activating transcription factor 3 (Atf3), early growth response 1 (Egr1), and runt related transcription factor 1 (Runx1) (Fig. 6(B)). This pathway enrichment was not conferred by IL-17 A, but by downstream transcription factor AP-1, Fosb and target inflammatory genes such as IL-6, Csf2, Ptgs2, and Cxcl1 (Fig. 6(C)). As shown in Fig. 6(D) and Fig. 4(B), these observations were confirmed by quantitative PCR (The relative ratio of *E*-KO mice to E-CT mice: [IL-17 A] 1.07 ± 0.14, *P* = 0.8092; [IL17RA] 0.99 ± 0.07, *P* = 0.8082; [IL17RC] 1.04 ± 0.03, *P* = 0.8082; [Fosb] 0.3 ± 0.04, *P* = 0.0046; [Csf2] 0.2 ± 0.02, *P* = 0.0048; [Cxcl1] 0.3 ± 0.02, *P* = 0.0048; [Cxcl10] 0.4 ± 0.08, *P* = 0.0123; [Ptgs2] 0.4 ± 0.02, *P* = 0.0048). These suggest a potential involvement of endothelial CXADR in IL-17 A-mediated inflammatory responses.

### 3.7 CXADR regulates the MKK3/6-p38-MAPK and p65/NF-κB pathways

**Fig. 7:**
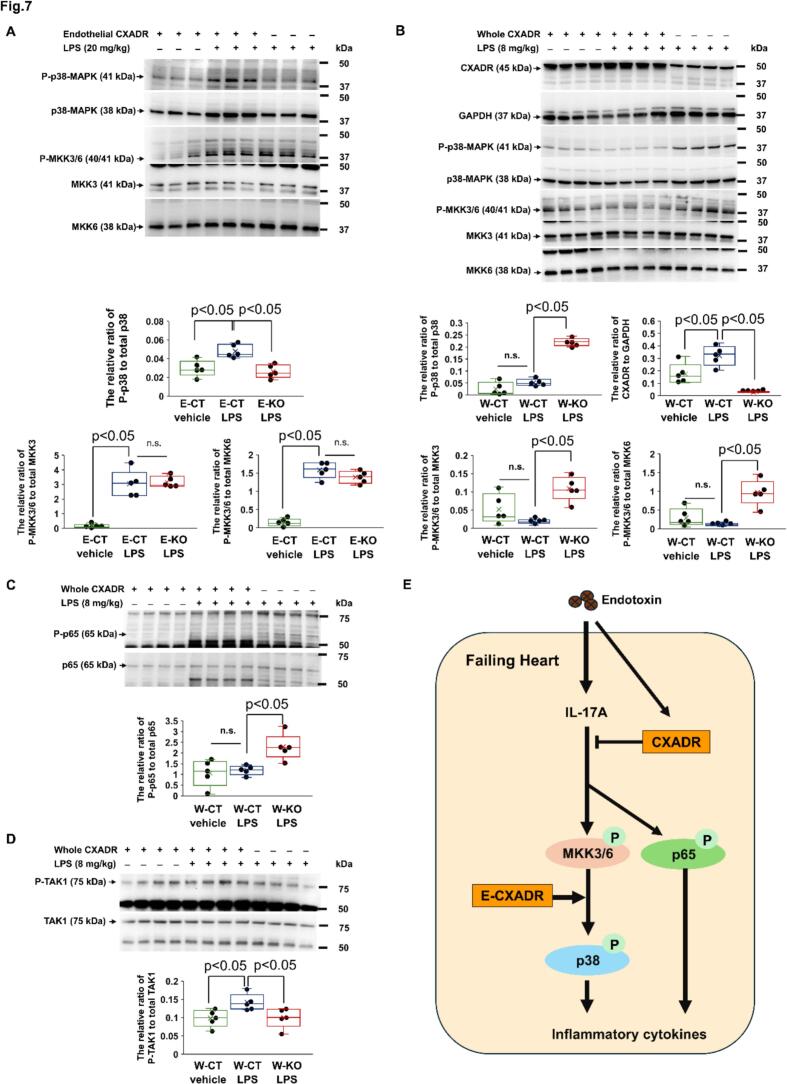
**The role of CXADR on the activation of MKK3/6-p38 and NF-κB/p65 in the failing heart after endotoxin challenge.**. (**A-D)** Protein expressions of phosphorylated p38 (P-p38), phosphorylated mitogen-activated protein kinase kinase 3/6 (P-MKK3/6), phosphorylated p65 (P-p65), and phosphorylated TGF-β-activated kinase1 (P-TAK1), as well as total p38, total MKK3/6, coxsackievirus and adenovirus receptor (CXADR), total p65, and total TAK1, in the heart were assessed by immunoblotting in endothelium-specific CXADR knockout (*E*-KO), whole-body CXADR knockout (W-KO), and the each control (E-CT and W-CT) mice after challenge with 20 or 8 mg/kg of lipopolysaccharide (LPS) or vehicle alone (*n* = 5 each). Bulk heart samples were collected 12 h after challenge. The full-length blots corresponding to Fig. 7A and Fig. 7B, C, and D are presented in Fig. S5 and Fig. S6, respectively. P-p38, P-MKK3/6, P-p65, P-TAK1, and CXADR protein levels were quantified by densitometry and expressed as a relative ratio to total p38, total MKK3/6, total p65, total TAK1, and GAPDH, respectively. Individual data are shown as dot plots of the box-and-whisker diagram. *P* < 0.05 and n.s. ‘not significant’, as determined by the Mann-Whitney *U* test. Three biological replicates were performed for each experiment. (**E**) Schema of the hypothetical mechanism by which CXADR regulates inflammatory response in endotoxin-induced failing heart. P: phosphorylation, IL: interleukin, E-CXADR: endothelial CXADR.

To identify potential mechanisms by which CXADR regulates the IL-17 A-mediated inflammatory responses underlying endotoxin-induced cardiac dysfunction, we examined the association of CXADR with p38-MAPK, a key driver of IL-17 A signaling [[37], [38], [39]], and MKK3 and MKK6, which activate the p38-MAPK [40]. The significantly increased protein expression of phosphorylated p38 (P-p38) in E-CT hearts after LPS challenge compared with vehicle alone challenge (The ratio of P-p38 to total p38: 0.048 ± 0.003 vs. 0.030 ± 0.004, *P* = 0.0216, respectively) was inhibited by the E-KO (The ratio of P-p38 to total p38: 0.026 ± 0.003, *P* = 0.0122 vs. E-CT mice) (Fig. 7(A)). Protein expression of phosphorylated MKK3/6 (P-MKK3/6) significantly increased after LPS challenge in E-CT hearts compared to expression after vehicle alone challenge (The ratio of P-MKK3/6 to total MKK3 or MKK6: 3.043 ± 0.409 vs. 0.136 ± 0.062, *P* = 0.0119; 1.585 ± 0.101 vs. 0.141 ± 0.048, *P* = 0.0119; respectively), but was not affected by the E-KO (The ratio of P-MKK3/6 to total MKK3 or MKK6: 3.161 ± 0.173, *P* = 0.8340; 1.388 ± 0.077, *P* = 0.1425 vs. E-CT mice; respectively) (Fig. 7(A)).

On the other hand, protein expression of P-p38 in W-CT hearts did not increase 12 h after LPS challenge compared to after the vehicle alone challenge (The ratio of P-p38 to total p38: 0.052 ± 0.008 vs. 0.025 ± 0.012, *P* = 0.0937, respectively), accompanied by increased protein expression of cardiac CXADR (The ratio of CXADR to GAPDH: 0.323 ± 0.037 vs. 0.174 ± 0.038, *P* = 0.0367, respectively). Subsequently, when its increase was prevented by the W-KO in adult mice (The ratio of CXADR to GAPDH: 0.029 ± 0.002, *P* = 0.0122 vs. W-CT mice), P-p38 protein expression after LPS challenge significantly increased (The ratio of P-p38 to total p38: 0.225 ± 0.007, *P* = 0.0122 vs. W-CT mice) (Fig. 7(B)). Consistently, P-MKK3/6 protein expressions in the heart after LPS challenge significantly increased in W-KO mice compared to W-CT mice (The ratio of P-MKK3/6 to total MKK3 or MKK6: 0.109 ± 0.016 vs. 0.018 ± 0.003, *P* = 0.0117 or 0.969 ± 0.163 vs. 0.114 ± 0.019, *P* = 0.0117, respectively) (Fig. 7(B)). Alternatively, we examined the protein expression of phosphorylated p65 (P-p65), also known as an IL-17 A-responsive transcription factor. Similarly to P-p38, its expression after LPS challenge significantly increased in W-KO mice compared to W-CT mice (The ratio of P-p65 to total p65: 2.279 ± 0.277 vs. 1.187 ± 0.098, *P* = 0.0122, respectively) (Fig. 7(C)). We further examined the activation of TAK1, an activator of both MKK3/6 and NF-κB [41,42]. Protein expression of phosphorylated TAK1 (P-TAK1) slightly increased in W-CT hearts after LPS challenge compared to after vehicle alone challenge (The ratio of P-TAK1 to total TAK1: 0.142 ± 0.010 vs. 0.099 ± 0.011, *P* = 0.0367, respectively). However, its increase was rather prevented in W-KO mice (The ratio of P-TAK1 to total TAK1: 0.100 ± 0.012, *P* = 0.0216 vs. W-CT mice) (Fig. 7(D)). Collectively, our data suggest that endothelial CXADR augments the activation of p38 by MKK3/6, whilst increased CXADR in endotoxin-induced failing heart attenuates the activation of MKK3/6 as well as NF-κB/p65, independently of TAK1 activation, ultimately inhibiting IL-17 A-mediated inflammatory responses (Fig. 7(E)).

## 4 Discussion

We demonstrated that CXADR has anti-inflammatory and cardioprotective effects in endotoxin-induced failing heart characterized by IL-17 A-mediated inflammatory responses, besides its role as an inflammatory mediator. Moreover, as a potential mechanism for these dual functions, we found that CXADR induces endothelium-mediated cell recruitment whilst also attenuating the activation of the MKK3/6-p38 and NF-κB/p65 pathways that drive the IL-17 A response responsible for cardiac dysfunction. The CXADR-MKK3/6-p38 and CXADR-p65 axes may represent a novel protective mechanism against inflammatory heart failure, whilst selectively inhibiting endothelial CXADR expression could yield clinical benefit.

IL-17 A-mediated inflammatory response appears to be a cornerstone of endotoxin-induced cardiac dysfunction. The IL-17 A has been demonstrated to play a pathological role in cardiac dysfunction derived from endotoxin stress and sepsis as well as ischemia, transplantation, hypertension, and autoimmunity [[43], [44], [45], [46], [47], [48], [49], [50]]. IL-17 A-induced inflammatory mediators, including IL-6, IL-1β, Ptgs2, Csf2, and Cxcl1, can cause myocardial inflammation and depression [[10], [11], [12],^14^], and have been associated with endotoxin-induced cardiac dysfunction and shock [14,[51], [52], [53], [54]]. Consistently, we identified the IL-17 A response in endotoxin-induced failing heart.

CXADR diversely modulates cardiac dysfunction characterized by the IL-17 A response. CXADR is a major endogenous ligand for junctional adhesion molecule-like proteins (JAML) expressed on γδ T cells, neutrophils, and monocytes [[22], [23], [24], [25]]. This molecular interaction has been shown to promote cell proliferation and cytokine production by activating signal transduction [55]. In the sepsis model, IL-17 A expression was reported to be derived from γδ T cells [56]. IL-17 A in turn stimulates the production of inflammatory cytokines and chemokines by endothelial and epithelial cells via p38 MAPK [37,38]. Thus, endothelial and epithelial CXADR-JAML interaction likely induces immune cell recruitment by promoting the IL-17 A signaling, resulting in inflammatory organ dysfunction. This is consistent with the histological and molecular biological findings observed in endothelial cell-specific CXADR-deficient mice, as well as with a previous observation showing that selectively depleting CXADR from epithelial cells inhibits the infiltration of immune cells into the lungs [29]. Moreover, in terms of CXADR-JAML interaction, mice overexpressing CXADR in cardiomyocytes also developed inflammatory cardiomyopathy, independent of viral infection [28]. On the other hand, in the present study, depleting CXADR from the whole heart of adult mice unexpectedly promoted cardiac pathological responses to endotoxin. This shows that CXADR exerts dual pro- and anti-inflammatory effects in endotoxin-induced cardiac dysfunction. Moreover, these contradictory findings suggest a cell type-specific role for CXADR in the heart.

Apart from its physical function as an intercellular adhesion molecule, we found that CXADR functions as a novel regulator of the MKK3/6-p38 MAPK and NF-κB/p65 pathways in endotoxin-induced cardiac dysfunction. The p38 MAPK has been demonstrated to drive the production of inflammatory cytokines by endothelial cells [37,38], cardiomyocytes, and cardiac fibroblasts in response to IL-17 A [57,58] and to mediate endotoxin-induced cardiac dysfunction [59,60]. Moreover, the activation of p38 MAPK is predominantly mediated through the upstream kinases, MKK3 and MKK6 [40]. It has been demonstrated that the pathological effects of inflammatory cytokines on myocardial contractility are elicited by the MKK3/6-activated p38-MAPK pathway [[61], [62], [63]]. Our data showed that localized CXADR in endothelial cells promotes the action of MKK3/6 on p38, whereas increased CXADR throughout the heart attenuates the activation of MKK3/6, ultimately inhibiting p38 MAPK. Additionally, increased CXADR in the heart also attenuated the activation of NF-κB, which drives the IL-17 A response and mediates endotoxin-induced cardiac dysfunction [64,65]. These observations suggest that CXADR differentially engages the MKK3/6-p38 pathway depending on its cellular origin, leading to the dual pro- and anti-inflammatory roles of CXADR in endotoxin-induced cardiac dysfunction.

CXADR may affect intracellular signaling via its intracellular domain [66]. To identify the mechanism by which CXADR regulates MKK3/6-p38 and NF-κB/p65 pathways, we examined phosphorylation of TAK1 which activates these pathways [41,42]. However, its phosphorylation was reduced by depleting CXADR. Meanwhile, MEKK3, which, like TAK1, belongs to the MAP3K family, can induce NF-κB activation, independently of TAK1 [67], and also links to the MKK3/6-p38 MAPK pathway [68]. Thus, assessing MEKK3 activation may allow for a more detailed characterization of the anti-inflammatory effect of CXADR. The regulation of heart-derived pathological intracellular signaling by CXADR will be an interesting research topic in the control of inflammatory heart failure.

### 4.1 Clinical implications

Whilst whole-body removal of CXADR promotes pathological cardiac responses, the protective effects of endothelium-specific CXADR knockout against myocardial inflammation, established cardiac dysfunction, shock, and subsequent death suggest the potential of selectively inhibiting endothelial CXADR expression as a therapeutic strategy for inflammatory heart disease. On the other hand, in view of the dual pro- and anti-inflammatory effects, increased CXADR in the heart may be beneficial in the treatment of infectious heart disease by eliminating pathogens while controlling aberrant inflammatory response. Moreover, overexpressing CXADR in cardiomyocytes has been reported to cause an inflammatory cardiomyopathy [27,28]. CXADR expression should be appropriately controlled in the heart. The targeted manipulation of CXADR expression would open new possibilities for therapeutic interventions in various heart diseases.

### 4.2 Study limitations

The present study has several limitations. Firstly, the presence of CXADR deletion prior to endotoxin challenge is impossible in the clinical setting of sepsis. Therefore, whether CXADR knock-down or deletion after the onset of disease would also provide similar protection requires further investigation. Secondly, while our model reflected the clinical and biological conditions of the septic heart [[2], [3], [4]], it did not reflect the widespread human septic pathology. Thirdly, bacterial replication and related systemic responses cannot be reproduced in our model. To define the effects of CXADR on these phenomena, testing in another sepsis model caused by cecum ligation and puncture is required [56]. Fourthly, the mechanism by which CXADR regulates heart-derived inflammatory responses was not fully elucidated. Intracellular signaling pathways need to be investigated using in vitro experimental systems involving endothelial cells and cardiomyocytes.

## 5 Conclusions

This study is the first to investigate the clinical and biological differences in whole-body and endothelium-specific conditional knockout mice of CXADR and to demonstrate its dual function as both a proinflammatory mediator and an anti-inflammatory protector in endotoxin-induced cardiac dysfunction. Moreover, we found that, possibly depending on the cellular origin, CXADR positively or negatively regulates the activation of cardiac p38, known to promote inflammatory cardiac dysfunction. This study suggest a novel involvement of CXADR in the regulation of pathological inflammatory response in the heart to endotoxin via p38, whilst highlighting the potential for targeted manipulation of CXADR expression as a therapeutic strategy for endotoxin-induced cardiac dysfunction.

## CRediT authorship contribution statement

**Reo Matsumura:** Investigation, Formal analysis. **Mototsugu Nishii:** Writing – original draft, Funding acquisition, Formal analysis, Data curation, Conceptualization. **Haruya Usuku:** Formal analysis. **Masahiro Nakayama:** Data curation. **Masaki Hachisuka:** Formal analysis. **Naho Misawa:** Formal analysis. **Ryo Saji:** Formal analysis. **Fumihiro Ogawa:** Methodology. **Alan Valaperti:** Supervision, Conceptualization. **Yoshihiro Ishikawa:** Supervision. **Ichiro Takeuchi:** Formal analysis.

## Contributions

M.N. and R.M. generated the hypothesis and experimental design. M.N. and R.M. prepared the manuscript. R.M., H.U., M.N., M.H., R.S., N.M., F.O., V.A., Y.I., and I.T. conducted experiments and helped with data analysis. All the authors reviewed and edited the manuscript.

## Funding

This work was supported by Grant-in-Aid for Scientific Research (MEXT/JSPS KAKENHI Grant Numbers JP19K09401, JP24K12203) and 10.13039/100008729Yokohama Foundation for Advancement of Medical Science (WAKABA research grants). These funding grants only provided financial support in the form of research materials.

## Declaration of competing interest

none declared.

## Data Availability

The data underlying this article will be shared upon reasonable request to the corresponding author.

## Appendix A Supplementary data

Supplementary material Image 1
