# Supplementary material for: Coxsackie and adenovirus receptor is a novel regulator of inflammatory response in endotoxin-induced failing heart
Source: J Mol Cell Cardiol Plus. 2025 Nov 6;14:100496. doi: 10.1016/j.jmccpl.2025.100496 (PMC12651843; doi:10.1016/j.jmccpl.2025.100496)
Supplement: Supplementary file 1 — Supplementary material [file mmc1.docx]

**Coxsackie and adenovirus receptor is a novel regulator of inflammatory response in endotoxin-induced failing heart.**

Reo Matsumura, MD ^1^, Mototsugu Nishii, MD, PhD ^1^*, Haruya Usuku ^1^, Masahiro Nakayama, MD ^1^, Masaki Hachisuka, MD ^1^, Naho Misawa, MD ^1^, Ryo Saji, MD, PhD ^1^, Fumihiro Ogawa, MD, PhD ^1^, Alan Valaperti, PhD ^2^, Yoshihiro Ishikawa, MD, PhD ^3^, Ichiro Takeuchi, MD, PhD ^1^

**Supplemental Figures 1 to 6**

**Supplemental Tables 1 to 4**

Short Title: CXADR in endotoxin-stressed heart

Original Articles, 5994 words

* Corresponding author: Mototsugu Nishii, MD, PhD

Email: s461211@yokohama-cu.ac.jp

Address: Department of Emergency Medicine, Yokohama City University Graduate School of Medicine, A102, 3-9 Fukuura, Kanazawa-ku, Yokohama, Kanagawa 236-0004, Japan.

**Supplemental Figures**

**Figure S1. Genotypes**

**
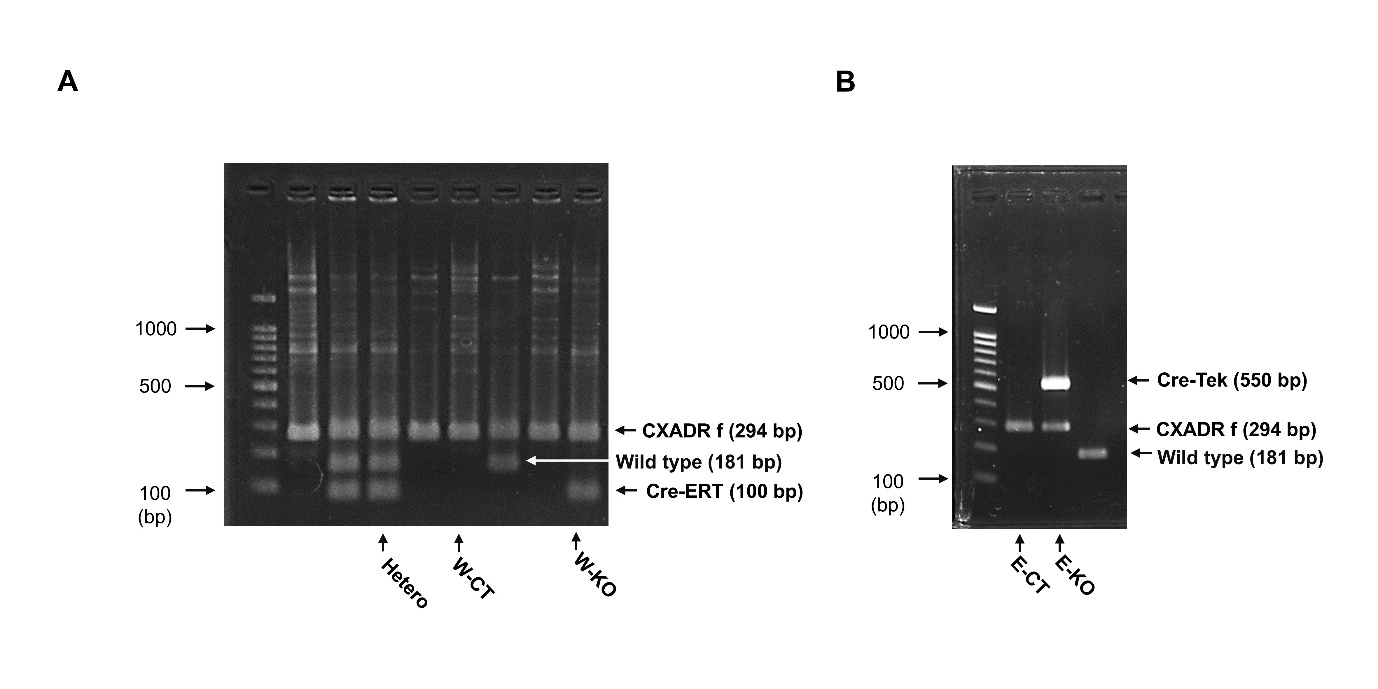
**

**A.** Genotypes of tamoxifen-induced whole-body coxsackie and adenovirus receptor (CXADR) knockout (W–KO) and control (W–CT) mice. W-KO was shown by genomic bands of both CXADR flox (f) and CRE-ERT, while W-CT or heterozygous genotype by CXADR f only or three bands of CXADR f, wild type, and CRE-ERT, respectively. **B**. Genotypes of endothelium-specific CXADR knockout (E–KO) and control (E–CT) mice. E-KO was shown by genomic bands of both CXADR f and CRE-Tek, while E-CT by CXADR f only.

**Figure S2. Full-length western blots**


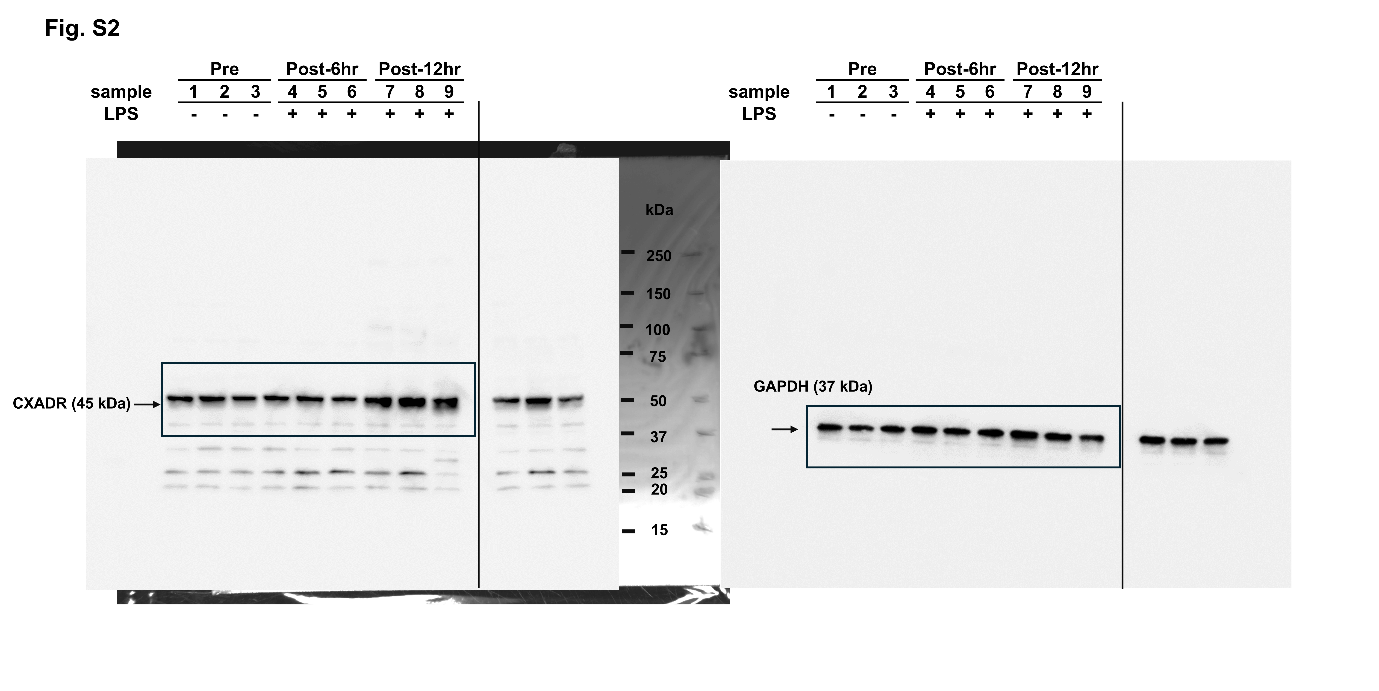


Representative full-length western blots of coxsackie and adenovirus receptor (CXADR) as well as GAPDH in bulk heart tissues from wild-type mice before and after challenge with 32 mg/kg lipopolysaccharide (LPS) are shown together with protein markers. Enhanced chemiluminescent images with ECL reagent were captured and quantified with the LAS-3000 luminescent image analyzer (Fujifilm, Co.).

**Figure S3. Full-length western blots**


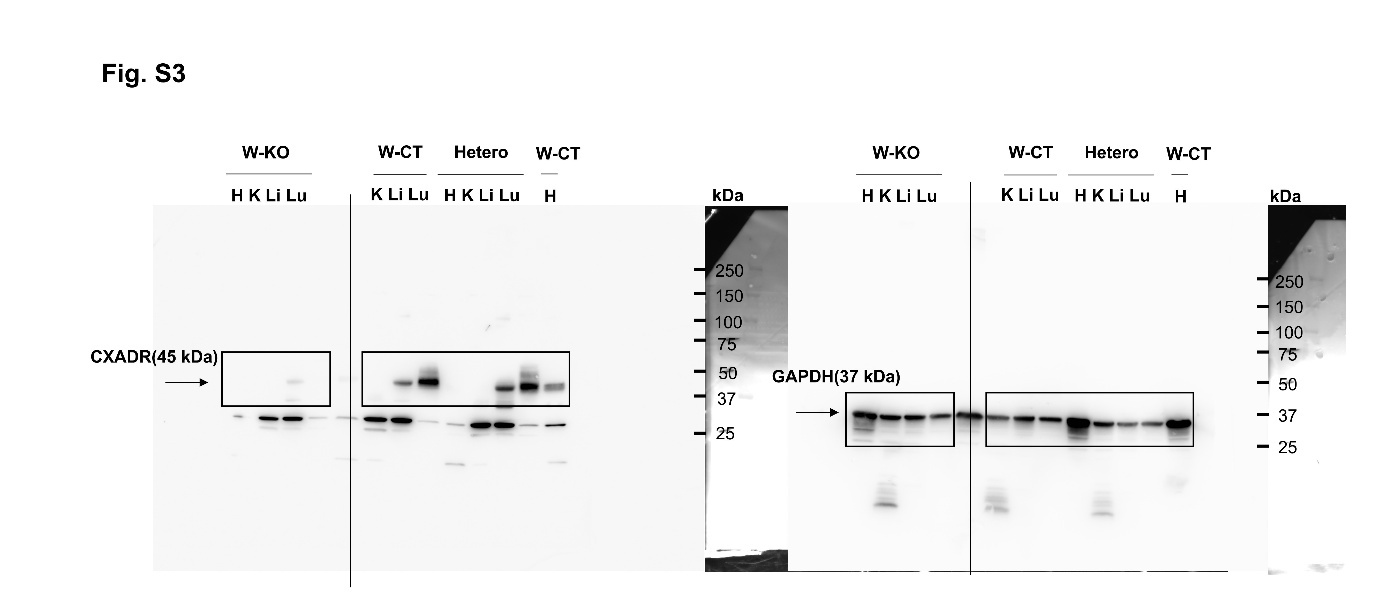


Representative full-length western blots of coxsackievirus and adenovirus receptor (CXADR) as well as GAPDH in lung (Lu), liver (Li), kidney (K), and heart (H) from whole-body CXADR knockout (W-KO), control (W-CT), and heterogynous type mice are shown together with protein markers. Enhanced chemiluminescent images with ECL reagent were captured and quantified with the LAS-3000 luminescent image analyzer (Fujifilm, Co.).

**Figure S4. Full-length western blots**


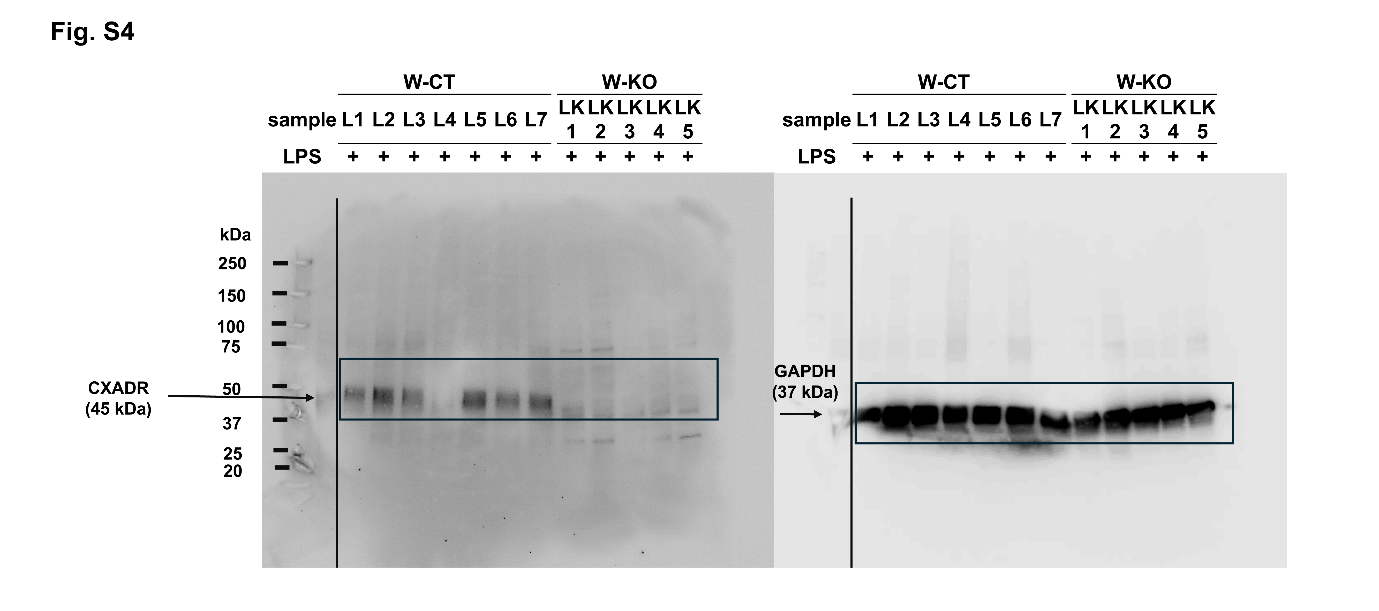
Representative full-length western blots of coxsackie and adenovirus receptor (CXADR) as well as GAPDH in bulk heart tissues from whole-body CXADR knockout (W-KO) and control (W-CT) mice 12 hours after challenge with 8 mg/kg of lipopolysaccharide (LPS) are shown together with protein markers. Sample numbers: L (LPS) and K (knockout). Enhanced chemiluminescent images with ECL reagent were captured and quantified with the LAS-3000 luminescent image analyzer (Fujifilm, Co.).

**Figure S5. Full-length western blots**


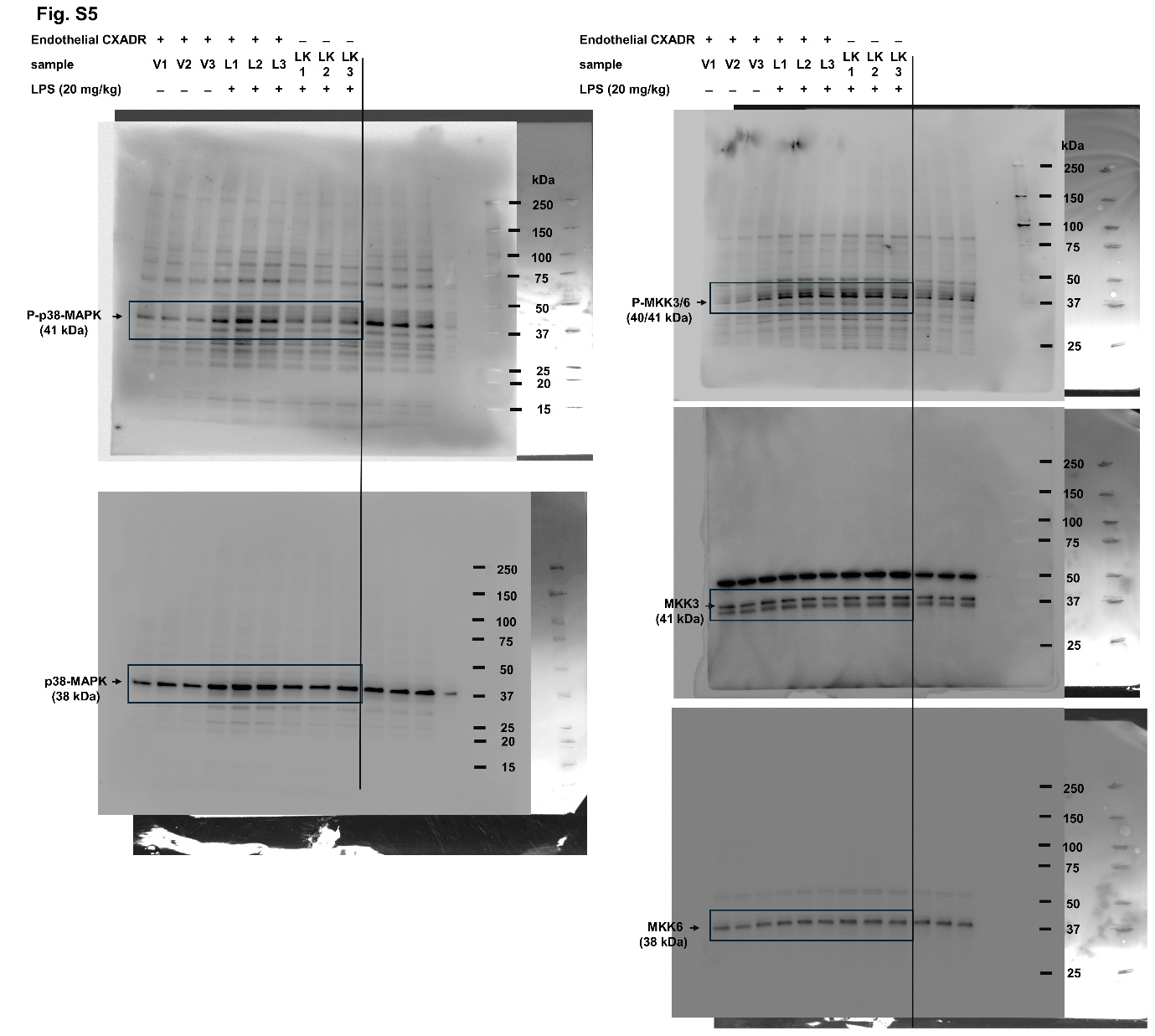


Representative full-length western blots of phosphorylated p38 (P-p38) and phosphorylated mitogen-activated protein kinase kinase 3/6 (P-MKK3/6) as well as total p38 and total MKK3/6 in bulk heart tissues from endothelium-specific CXADR knockout and control mice 12 hours after challenge with 20 mg/kg of lipopolysaccharide (LPS) or vehicle alone are shown together with protein markers. Sample numbers: V (vehicle alone), L (LPS), and K (knockout). Enhanced chemiluminescent images with ECL reagent were captured and quantified with the LAS-3000 luminescent image analyzer (Fujifilm, Co.).

**Figure S6. Full-length western blots**

**
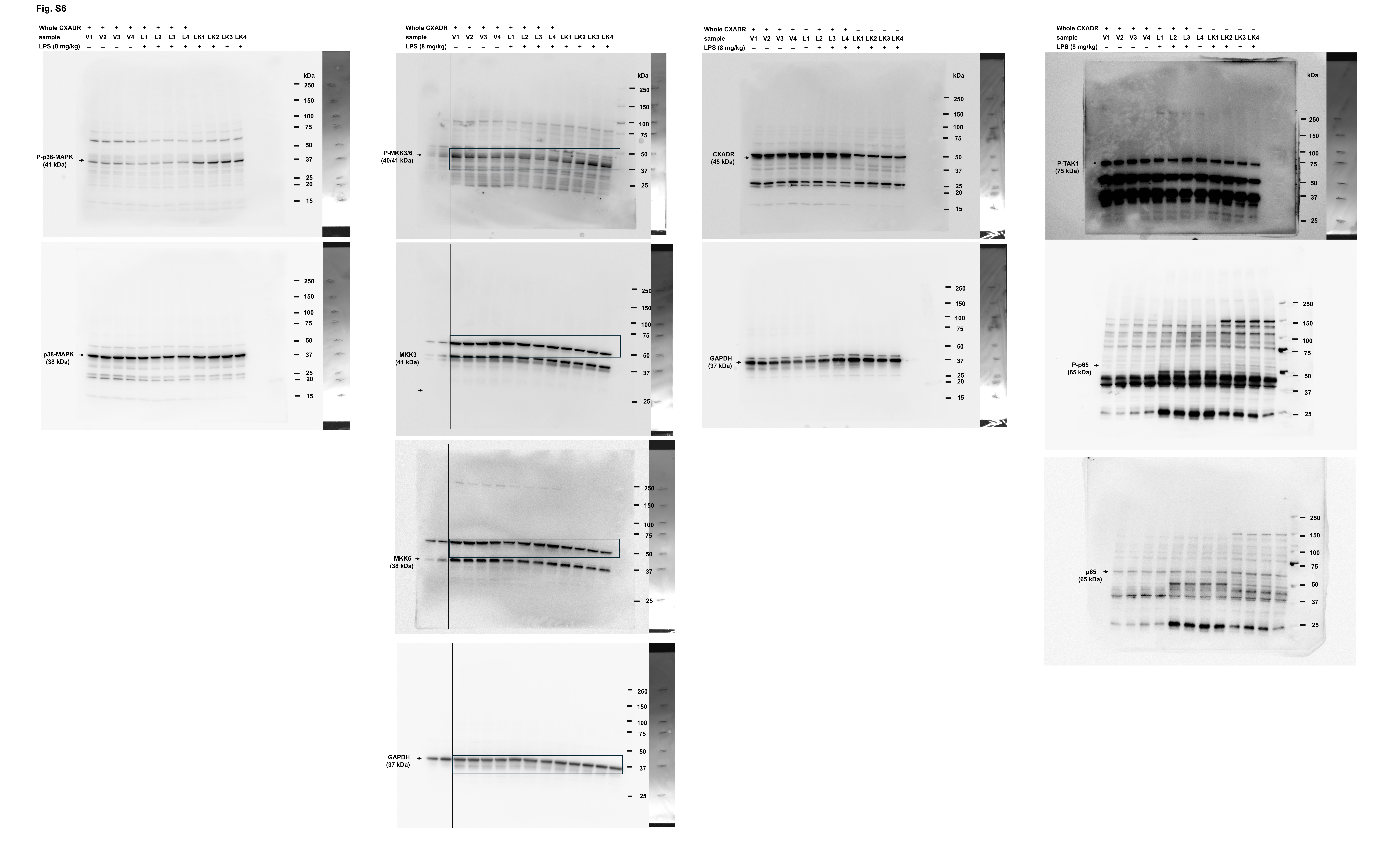
**

Representative full-length western blots of phosphorylated p38 (P-p38), phosphorylated mitogen-activated protein kinase kinase 3/6 (P-MKK3/6), coxsackievirus and adenovirus receptor (CXADR), phosphorylated p65 (P-p65), and phosphorylated TGF-β-activated kinase1 (P-TAK1) as well as total p38, total MKK3/6, total p65, and GAPDH in bulk heart tissues from whole-body CXADR knockout and control mice 12 hours after challenge with 8 mg/kg of lipopolysaccharide (LPS) or vehicle alone are shown together with protein markers. Sample numbers: V (vehicle alone), L (LPS), and K (knockout). Enhanced chemiluminescent images with ECL reagent were captured and quantified with the LAS-3000 luminescent image analyzer (Fujifilm, Co.).

**Supplemental Tables**

**Table S1. Sequences of primers**

| Gene | Sense | | | Antisense |
| --- | --- | --- | --- | --- |
| Real-time PCR |  |  |  |  |
| CXADR | CTCTGACCAGTGTATGCTGCGA | | | CTCTGCGTTTCCTGTGACAGCA |
| IL-6 | TGTTCTCTGGGAAATCGTGGA | | | CTGCAAGTGCATCATCGTTGT |
| IL-1β | CAACCAACAAGTGATATTCTCCATG | | | GATCCACACTCTCCAGCTGCA |
| BNP | ATGGATCTCCTGAAGGTGCTG | | | GTGCTGCCTTGAGACCGAA |
| IL-17A | TTTAACTCCCTTGGCGCAAAA | | | CTTTCCCTCCGCATTGACAC |
| IL-17RC | GAGTCCCTGCCAGCCACTT | | | ACTGGAAATCTTGTGGCTCATTC |
| IL-17RA | CCCAGTAATCTCAAATACCACAGTTC | | | CGATGAGTGTGATGAGGCCATA |
| Cxcl1 | CTTGAAGGTGTTGCCCTCAG | | | TGGGGACACCTTTTAGCATC |
| Cxcl10 | ATCATCCCTGCGAGCCTATCCT | | | GACCTTTTTTGGCTAAACGCTTTC |
| Csf2 | TCAAAGAAGCCCTGAACCTCC | | | GTGAAATTGCCCCGTAGACC |
| Ptgs2 | GCGACATACTCAAGCAGGAGCA | | | AGTGGTAACCGCTCAGGTGTTG |
| Fosb | GTGAGAGATTTGCCAGGGTC | | | AGAGAGAAGCCGTCAGGTTG |
| Fos | GGAATTAACCTGGTGCTGGA | | | TGAACATGGACGCTGAAGAG |
| Nfkb1 | GCTGCCAAAGAAGGACACGACA | | | GGCAGGCTATTGCTCATCACAG |
| GAPDH | GTGTTCCTACCCCCAATGT | | | TGTCATCATACTTGGCAGGTTTC |
| Genotyping PCR | |  |  |  |
| CXADR | GGTGTGATGTTAGTGAGGAACG | | | CTGCTCCAGATTCCCACAAT |
| Tek cre | CCCTGTGCTCAGACAGAAATGAGA | | | CGCATAACCAGTGAAACAGCATTGC |
| Cre ER | GCGGTCTGGCAGTAAAAACTATC | | | GTGAAACAGCATTGCTGTCACTT |

CXADR: Coxsackie and adenovirus receptor; IL: interleukin; BNP: B-type natriuretic peptide; Cxcl: C-X-C motif chemokine ligand; Csf: Colony stimulating factor; TNF: Tumor necrosis factor.

| Sample | RNA（ng） | RIN |
| --- | --- | --- |
| E-CT1 | 9,957 | 9.4 |
| E-CT2 | 14,992 | 9.4 |
| E-CT3 | 8,951 | 9.5 |
| E-KO1 | 8,061 | 9.2 |
| E-KO2 | 7,792 | 9.1 |
| E-KO3 | 5,015 | 9.3 |
| Sham1 | 5,491 | 9.3 |
| Sham2 | 3,385 | 9.5 |
| Sham3 | 4,937 | 9.3 |

**Table S2. The quality of isolated RNA**

Endothelium-specific coxsackievirus and adenovirus receptor knockout mice (E-KO) and control mice (E-CT) were intraperitoneally given lipopolysaccharide at 20 mg/kg, while sham mice vehicle only. Heart tissue was collected at 12 hours post-challenge for Bulk RNA sequencing. The quality of isolated RNA was evaluated by the TapeStation system using High Sensitivity RNA ScreenTape (Agilent). RIN: RNA integrity number.

**Table S3. Changes over time in echocardiographic data in** **tamoxifen-induced whole-body CXADR knockout mice**

| **Variables** |  | **Changes over time** | | |  | ***P*** | | | | |
| --- | --- | --- | --- | --- | --- | --- | --- | --- | --- | --- |
|  |  | **Pre-challenge** | **6 hours** | **12 hours** |  | **ANOVA** |  | **Post hoc** | | |
|  |  |  |  |  |  |  |  | **G1 vs. G2** | **G1. vs G3** | **G2 vs. G3** |
| **W-CT/Vehicle (n = 5)** |  |  |  |  |  |  |  |  |  |  |
| HR (bpm) |  | 663.7±18.31 | 677.4±8.33 | 659.0±15.25 |  | 0.6764 |  |  |  |  |
| LVFS (%) |  | 63.7±2.20 | 64.6±1.20 | 63.8±3.00 |  | 0.9099 |  |  |  |  |
| LVDd (mm) |  | 2.6±0.09 | 2.9±0.12 | 2.7±0.20 |  | 0.2512 |  |  |  |  |
| LVSd (mm) |  | 0.95±0.07 | 1.03±0.05 | 0.99±0.15 |  | 0.8482 |  |  |  |  |
| **W-KO/Vehicle (n = 5)** |  |  |  |  |  |  |  |  |  |  |
| HR (bpm) |  | 663.4±6.47 | 656.6±12.80 | 669.0±18.05 |  | 0.7866 |  |  |  |  |
| LVFS (%) |  | 64.3±2.06 | 64.7±3.83 | 67.2±3.70 |  | 0.8232 |  |  |  |  |
| LVDd (mm) |  | 2.5±0.08 | 2.8±0.12 | 2.6±0.12 |  | 0.1575 |  |  |  |  |
| LVSd (mm) |  | 0.90±0.03 | 0.99±0.14 | 0.87±0.12 |  | 0.7719 |  |  |  |  |
| **W-CT/LPS (n = 14)** |  |  |  |  |  |  |  |  |  |  |
| HR (bpm) |  | 639.4±8.73 | 579.4±11.94 | 447.0±8.77 |  | <0.0001 |  | 0.0002 | <0.0001 | <0.0001 |
| LVFS (%) |  | 64.0±1.45 | 42.7±2.98 | 46.0±2.18 |  | <0.0001 |  | <0.0001 | <0.0001 | 0.5706 |
| LVDd (mm) |  | 2.6±0.07 | 2.5±0.08 | 2.0±0.05 |  | <0.0001 |  | 0.1398 | <0.0001 | <0.0001 |
| LVSd (mm) |  | 1.0±0.06 | 1.4±0.10 | 1.1±0.06 |  | 0.0002 |  | 0.0002 | 0.6252 | 0.0033 |
| **W-KO/LPS (n = 8)** |  |  |  |  |  |  |  |  |  |  |
| HR (bpm) |  | 666.4±11.97 | 624.3±19.91 | 518.0±17.04 |  | <0.0001 |  | 0.0134 | <0.0001 | 0.0008 |
| LVFS (%) |  | 63.1±2.28 | 38.5±4.68 | 29.2±4.95 |  | <0.0001 |  | 0.0007 | <0.0001 | 0.2871 |
| LVDd (mm) |  | 2.6±0.06 | 2.2±0.11 | 2.1±0.17 |  | 0.1231 |  |  |  |  |
| LVSd (mm) |  | 0.9±0.07 | 1.4±0.13 | 1.5±0.16 |  | 0.0153 |  | 0.0375 | 0.0152 | 0.9102 |

Data are mean ± SEM using the indicated number of mice. Echocardiographic findings were assessed before and 6 and 12 h (hours) after challenge with lipopolysaccharide (LPS) or vehicle alone in tamoxifen-induced whole-body coxsackie and adenovirus receptor (CXADR) knockout mice (W–KO) and control mice (W–CT). *P* values were calculated with two-way ANOVA followed by Dunnett test (vs. pre-challenge) and Tukey’s HSD test (6 vs. 12 hours). G1: pre-challenge; G2: 6 hours; G3: 12 hours; HR: heart rate; LVFS: left ventricular fractional shortening; LVDd: LV diastolic dimension; LVSd: LV systolic dimension.

**Table S4. Changes over time in echocardiographic data in endothelium-specific CXADR knockout mice**

| **Variables** |  | **Changes over time** | | |  | ***P*** | | | | |
| --- | --- | --- | --- | --- | --- | --- | --- | --- | --- | --- |
|  |  | **Pre-challenge** | **6 hours** | **12 hours** |  | **ANOVA** |  | **Post hoc** | | |
|  |  |  |  |  |  |  |  | **G1 vs. G2** | **G1. vs G3** | **G2 vs. G3** |
| **E-CT/Vehicle (n=5)** |  |  |  |  |  |  |  |  |  |  |
| HR (bpm) |  | 656.5±32.70 | 647.5±13.77 | 696.7±5.58 |  | 0.7089 |  |  |  |  |
| LVFS (%) |  | 62.1±2.39 | 62.3±1.96 | 62.8±2.27 |  | 0.7912 |  |  |  |  |
| LVDd (mm) |  | 2.7±0.05 | 2.5±0.06 | 2.6±0.13 |  | 0.8482 |  |  |  |  |
| LVSd (mm) |  | 1.0±0.07 | 0.9±0.07 | 1.0±0.09 |  | 0.8964 |  |  |  |  |
| **E-KO/Vehicle (n=5)** |  |  |  |  |  |  |  |  |  |  |
| HR (bpm) |  | 647.5±8.62 | 657.5±10.77 | 686.7±7.58 |  | 0.7232 |  |  |  |  |
| LVFS (%) |  | 61.0±1.74 | 62.3±2.06 | 62.5±2.07 |  | 0.6625 |  |  |  |  |
| LVDd (mm) |  | 2.8±0.10 | 2.6±0.06 | 2.5±0.07 |  | 0.8719 |  |  |  |  |
| LVSd (mm) |  | 1.1±0.07 | 1.0±0.10 | 0.9±0.03 |  | 0.8866 |  |  |  |  |
| **E-CT/LPS (n=8)** |  |  |  |  |  |  |  |  |  |  |
| HR (bpm) |  | 676.2±18.51 | 425.3±9.76 | 501.0±25.80 |  | 0.0023 |  | <0.0001 | 0.0001 | 0.0410 |
| LVFS (%) |  | 62.7±4.17 | 19.5±1.75 | 11.2±0.91 |  | <0.0001 |  | <0.0001 | <0.0001 | 0.0160 |
| LVDd (mm) |  | 2.7±0.08 | 3.3±0.09 | 3.8±0.12 |  | <0.0001 |  | 0.0074 | <0.0001 | 0.0140 |
| LVSd (mm) |  | 1.0±0.11 | 2.6±0.06 | 3.4±0.13 |  | <0.0001 |  | <0.0001 | <0.0001 | 0.0010 |
| **E-KO/LPS (n=8)** |  |  |  |  |  |  |  |  |  |  |
| HR (bpm) |  | 647.5±8.62 | 468.1±10.14 | 573.6±21.05 |  | 0.0002 |  | 0.0001 | 0.0385 | 0.0037 |
| LVFS (%) |  | 61.0±1.74 | 16.1±1.46 | 23.1±1.21 |  | <0.0001 |  | <0.0001 | <0.0001 | 0.0070 |
| LVDd (mm) |  | 2.8±0.10 | 3.4±0.09 | 3.1±0.05 |  | 0.0006 |  | 0.0003 | 0.0434 | 0.0152 |
| LVSd (mm) |  | 1.1±0.07 | 2.8±0.07 | 2.3±0.06 |  | <0.0001 |  | <0.0001 | <0.0001 | 0.0002 |

Data are mean ± SEM using the indicated number of mice. Echocardiographic findings were assessed before and 6 and 12 h (hours) after challenge with lipopolysaccharide (LPS) or vehicle alone in endothelium-specific coxsackie and adenovirus receptor (CXADR) knockout mice (E–KO) and control mice (E–CT). *P* values were calculated with two-way ANOVA followed by Dunnett test (vs. pre-challenge) and Tukey’s HSD test (6 vs. 12 hours). G1: pre-challenge; G2: 6 hours; G3: 12 hours; HR: heart rate; LVFS: left ventricular fractional shortening; LVDd: LV diastolic dimension; LVSd: LV systolic dimension.
